# Measuring education in the context of health inequalities

**DOI:** 10.1093/ije/dyac058

**Published:** 2022-04-01

**Authors:** Saman Khalatbari-Soltani, Janet Maccora, Fiona M Blyth, Camille Joannès, Michelle Kelly-Irving

**Affiliations:** The University of Sydney School of Public Health, Faculty of Medicine and Health, Sydney, NSW, Australia; ARC Centre of Excellence in Population Aging Research (CEPAR), University of Sydney, Sydney, Australia; ARC Centre of Excellence in Population Aging Research (CEPAR), University of Sydney, Sydney, Australia; School of Psychology, University of New South Wales, Sydney, New South Wales, Australia; The University of Sydney School of Public Health, Faculty of Medicine and Health, Sydney, NSW, Australia; ARC Centre of Excellence in Population Aging Research (CEPAR), University of Sydney, Sydney, Australia; Equity Research Team, CERPOP, Université de Toulouse, Inserm, Université Paul Sabatier, Toulouse, France; Equity Research Team, CERPOP, Université de Toulouse, Inserm, Université Paul Sabatier, Toulouse, France; Institut Fédératif d’études et Recherche Interdisciplinaire Santé Société (Iferiss), Université Toulouse III Paul Sabatier, Toulouse, France

## Introduction

Socio-economic inequalities in a wide range of health outcomes are pervasive and enduring.^1^ Most often, the association between socio-economic indicators and health is inversely graded (commonly known as social gradients in health) so that the higher the socio-economic position (SEP), the lower is the rate of morbidity and mortality. SEP is a broad concept capturing resource- and prestige-based measures.^2^ To date, educational level/attainment has been commonly used as an indicator of SEP in health inequalities research due to general acceptance that education is easy to measure and unlikely to be affected by diseases that begin in adult life.^3^ Of note, early-life health gradients in education (i.e. health selection hypothesis or reverse causality) might also exist and needs to be considered.^4^ Education is less prone to non-response error compared with income or wealth as SEP indicators.^3^ In most nations, education shapes the future occupational position and earning potential of individuals. However, it is important to keep in mind that in this context, education is only a ‘proxy’ or a probabilistic indicator of social class, status or income.^3^

**Figure 1:**
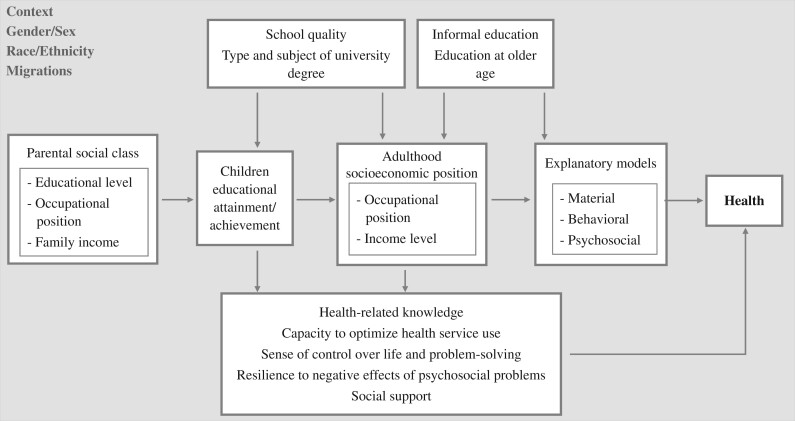
Pathways between education and health

Despite perceptions of education as a straightforward measure for social epidemiological purposes, the relationship between education and health is complex, as are the underlying mechanisms, including psychosocial, material and behavioural mechanisms and pathways (Figure 1).^3^ The psychosocial pathway emphasizes the way in which education provides a means to alleviate the direct and indirect effects of stress related to being lower on the socio-economic hierarchy through psychological and social buffering. The material pathway emphasizes that highly educated adults are more likely to be employed and earn more money, which improves access to tangible material conditions, services and amenities, and assets. The behavioural pathway emphasizes that education can increase health-related knowledge through health literacy, but also provides increased personal control, which in turn can lead to the adoption of healthy behaviours. These pathways are not mutually exclusive. The material resources of daily life across the life course have psychosocial meaning attached to them, one’s experience of psychosocial stress may be associated with health behaviours, etc. Whereas newer advanced epidemiological techniques and wider data availability increase the potential to improve understanding of the association between education and health and its underlying mechanisms, current practices regarding the measurement and operationalization of education as a core indicator of SEP lack the ability to consistently capture and compare the diversity of individual participants’ educational experiences over time.

Evidence is emerging that traditional education measures (educational qualification or years of schooling) are insufficient to fully understand the relationship between education and health. These traditional measures only focus on quantity and/or credentials and do not capture other facets of education, including the type and quality of education, school environment and dissonant/consonant relationships between family, and school and educational expectations. Traditional measures, mainly assessing early-childhood/adulthood educational experiences, typically neglect the importance of educational experiences throughout the life course based on assuming that education is unlikely to alter after early adulthood. Moreover, the justification for a specific conceptualization of education is often not adequately described. Of note, the choice of education as an SEP indicator and its operationalization often reflect the availability of data rather than any explicit theorization of the possible effect of different measures or categorizations. To further discussion regarding some of the problems with the current approaches to educational measurement in social inequality studies,^5–7^ our paper takes a different approach by both highlighting the methodological issues and emphasizing the importance of commonly neglected aspects of education in these studies.

## Educational measures

A widely used measure of education is based on attainment of a qualification or completion of a phase of education, such as primary or high school. Statistically, attainment measures can be dichotomized as having achieved a particular level or not or compared across categories of educational attainment. Some studies convert attainment measures into duration measures by allocating a number of years to each attainment level (e.g. finishing high school is commonly considered to be the equivalent of 12 years of education).

Another frequently used education measure is the duration, usually elicited as the number of years and sometimes as the age of leaving school. Years of education has the advantage of being easy to obtain and also offers flexibility for statistical manipulation. It is handled most elegantly and parsimoniously as a continuous variable but additionally lends itself to dichotomous and categorical operationalizations.^8^ Both attainment and duration measures can be relatively straightforward to acquire in narrow contexts with well-defined educational structures; however, they become more complex when there is ambiguity in educational pathways, especially when comparing across countries.^9^^,^^10^

Literacy measures, which reflect skills obtained through education, are also used depending on the context.^11^ Literacy can be self-reported or assessed directly.^11^ Other less commonly used educational measures include latent variable models—using multiple variables to evaluate a construct that cannot be measured directly (e.g. quality of education, academic achievement).^12^Table 1 summarizes different educational measures.

**Table 1:** Different educational measures

	Duration measures	Attainment measures	Literacy measures	Latent variable measures
Examples	Number of years, age leaving school	Qualification, completion of educational level	Reading level	Combination
Method of attainment	Primarily self-report or proxy, could be obtained from records, can be extrapolated from attainment measures	Primarily self-report or proxy, could be obtained from records	Self-report or testing	Could be combination of self-report or proxy, records, testing
Life-course stage	Primarily early life: childhood and adolescence	Primarily early life: childhood, adolescence and early adulthood	All stages up until that point	Throughout life course
Operationalization options dependent on the construct	Continuous, dichotomous, categorical	Dichotomous, categorical,conversion to continuous duration measure	Continuous, dichotomous, categorical	–
Ease of standardization across contexts	Simple to standardize	Complex to standardize	Moderately complex to standardize	Complex to standardize
Validity	Questionable	Moderate	High	Should be moderate to high

## Methodological issues regarding current approaches to measuring education

### All years are not created equal

With many continuous variables, we can expect each unit to be of relatively equal worth, even if there is a cumulative effect. This is not the case with education.^13^ A year of education at age 5 years cannot be expected to be comparable with a year of education at age 15 years. Similarly, a year of education in 1950 likely differs from a year of education in 2020,^14^ as does a year of education in Syria compared with a year in Sweden. A year of education in a classroom of 50 students is unlikely to be equivalent to a year of education in a classroom of 5. Moreover, using simple counts of years of education as a measure treats all types of education and educational institutions equally. Educational content varies across different subjects, thus a year in an art college is not equal to a year in an engineering school or a year in military academies.^14–16^ These are all relatively innocuous examples, but we must also consider that these inequalities in a year of education are likely to mirror existing inequalities in society in terms of gender, race/ethnicity, socio-economic and other (dis)advantages.^17^

A continuous measure of years of education assumes that every year of education contributes equally to a person’s acquired SEP and that time spent in education is more important than educational achievements.^13^ Due to differences in the structure and organization of courses across educational institutions and countries, it is possible that qualifications with very different levels of complexity require a similar amount of time in education.^15^ Consequently, measurement of the number of years of education of an individual is not necessarily a good proxy for educational attainment.^15^

### Self-reported educational level may not be reliable

In most cohort studies and surveys educational level is measured using a self-completed questionnaire.^7^^,^^18^ Although few studies have focused on the accuracy of reported education, there is some evidence of inconsistency in reporting educational attainment.^14^^,^^18^ Limited evidence also shows that respondents tend to exaggerate the amount of schooling they have obtained.^14^

### Inconsistencies in education measures

Consistency in measuring and modelling education allows researchers to compare the results from different studies and pool different data sources for within-country or cross-national comparative analysis.^19^ However, systematic reviews in the field highlight substantial heterogeneity in measuring and operationalizing education.^20^^,^^21^ Educational level does not have universal definitions. One important example in this regard is the definition of low education where researchers use different cut-offs for years of education to define low education, ranging from zero to 12.^20^ Importantly, results from studies that use different definitions are not comparable.

Comparability is particularly essential for cross-national studies.^10^ For cross-national comparison, educational data need to be harmonized using an internationally comparable education variable.^19^ Although attempts have been made to implement standardized comparative measures (e.g. harmonization using the International Standard Classification of Education), different studies have used either different standards or implemented them in different ways.^8^^,^^10^^,^^19^ Consistency is also important within the same context over time given the potential demographic changes and alterations in the educational system.^10^

Another important issue that makes comparability generally more difficult is the absence of explicit information about measurement of education and its operationalization. Information on how educational attainment was obtained (self-report, proxy, records), how the number of years were obtained (self-report, extrapolated), how categorization was determined (related to attainment, to years or to literacy) and what was the basis of cut-offs (educational system, population mean, literature, arbitrary) should all be reported but rarely are in practice.^20^

### Using education as a proxy for other concepts

Measurement of education as an SEP indicator is believed to capture the knowledge-related assets of an individual.^13^ Educational attainment also captures aspects of social opportunities for education and individual life chances.^19^ Moreover, educational attainment is strongly associated with attitudes, beliefs, values and behaviours as well as parents’ choices and constraints over how they can influence their children’s SEP.^19^^,^^22^ These highlight that education is a proxy for cultural capital, also captured by other variables such as the number of books in a child’s home.^22^^,^^23^ Some researchers argue that educational level is a proxy for intelligence that further affects health and health inequality^24^ despite findings from several studies that differences in educational achievements reflect the level of cultural capital and not intelligence.^25^^,^^26^ When interpreting results, it is important to distinguish between these two underlying hypothesized constructs.

## Specific considerations

### Birth cohort effects in education

Education systems change and evolve in most nations over time. Thus, the meaning of educational levels and implications of educational achievements vary among different birth cohorts.^13^ For instance, comparing educational levels of participants who attended school before or during the First World War with those who attended school after the Second World War is complicated as they have been educated under different circumstances with varying access to educational opportunities beyond compulsory education.^15^ Moreover, most of today’s older adults in Europe and the UK left school at the minimum age with no academic qualifications; thus, older cohorts will be over-represented among those classified as having low education.^27^ When using duration measures, researchers should consider changes to the length of compulsory education over time.^27^ Therefore, studies that include participants from several birth cohorts should consider specific birth cohort effects to avoid bias in their results.

### Gender/sex

From 1970 onwards, gender/sex differences in educational attainment levels, favouring male over female, have declined sharply in nearly all high-income countries (HICs) and, in some, have been eliminated or even reversed.^28^ However, the socio-economic impact of education on health is still gender-related; the education–occupation–wage relationship continues to favour men, with the economic returns for a given level of education higher for men than for women.^2^

Nevertheless, based on the resource substitution theory, educational level may have a greater impact on the health of women than men.^5^^,^^29^ This theory highlights that the less there is of one resource (e.g. earnings, power), the more important another will be (e.g. education), or the presence of one resource makes the absence of another less harmful.^29^ This impact on health has been observed with differences in mortality by educational level across age groups that are more pronounced in men than in women.^30^ Of note, it is important to highlight that maternal and paternal education might have different impacts on their offspring health. For instance, a 2021 global systematic review and meta-analysis highlighted that maternal education is a stronger predictor of child mortality than paternal education.^31^

### Race and ethnicity and migrants

The interactions between race, ethnicity and SEP should always be considered in interpreting the underlying mechanisms involved in education inequalities in health for several reasons. First, it has been shown that educational attainment is generally lower among ethnic minority populations.^32^ Apart from a lack of English-language proficiency, another important reason why ethnic minorities may have lower attendance or completion rates could be a lack of cultural competence in dominant educational paradigms (e.g. imposing cultural values and ignoring ancestral knowledge). Moreover, ethnic minority populations tend to receive a different amount of education than privileged population groups in a society and the quality of education received may also differ. Additionally, racial segregation in schools along with residential racial segregation or disproportional allocation of ethnic minority students into low-ability or non-college preparatory classes are primary mechanisms underlying socio-economic differences for many ethnic minority groups.^32^ It has been shown that sometimes attainment or duration measures are less appropriate than other measures for specific ethnic groups, likely due to differences in the quality of educational offerings. For instance, studies suggest that the quality of education is a more important consideration among minoritized people rather than years spent in education.^33^^,^^34^ Moreover, in some lower- and middle-income countries (LMICs) where compulsory education is either non-existent or not binding, just the fact of an adolescent attending school (or not) might be more relevant.

Third, ethnic minorities do not experience the same returns (e.g. economic returns) from education as dominant groups (the diminishing returns hypothesis).^18^ These differences are not only due to variations in educational quality, but also due to structural inequalities in society.^35^ Thus, weaker educational gradients among ethnic minorities, particularly women, could be due to smaller economic returns from education among these groups than privileged population groups.^5^ Finally, the extent to which education reflects socio-economic wellbeing may depend on the proportions of immigrants in different ethnic communities and whether they obtained their education outside or inside their host country, as the value attached to qualifications is often country-specific.^13^^,^^36^

### Nation-specific

Most SEP measures in common usage, including education, are developed by researchers from HICs.^13^^,^^18^ It is therefore important to question the appropriateness of imposing educational measures from HIC, with their accompanying cultural and values systems, on LMICs. Educational systems, with their idiosyncratic institutions and certificates and differential funding between public and private schools, differ substantially across countries, so common measures of education and their operationalization might not be appropriate in different contexts.^19^ This highlights the importance of using nation/country-specific educational measures in health inequality studies. Not paying attention to these important contextual details prevents us from capturing health inequalities by educational level. However, there is a conflict between the need for standardization for comparability and country-level appropriateness of educational measures. Researchers need to decide which is more appropriate for their research question and perhaps sacrifice one or the other, accepting and acknowledging the limitations in order to interpret their findings in this context. Researchers also need to account for characteristics specific to different populations. For example, reverse causality can be a factor, with the limiting effect of ill health in childhood on educational attainment a possible important consideration in LMIC.^11^ Of note, cohort effects and gender differences are likely to be of particular importance in LMIC compared with HICs.

## Neglected aspects of education

### School quality and environment

Currently, little is known about specific elements of education that might influence health. Measuring the number of years of education or levels of attainment captures no information about the quality of the education and/or school (e.g. pupil-to-teacher ratio, teacher salary, highly skilled teachers). For instance, school quality at critical time points in life (e.g. during adolescence) may influence long-term health trajectories.^37^ Dissonant/consonant relationships between family and school can be also an important factor in understanding the effects of education on health. In several studies, parental interest in their child’s education has been reported as an upstream determinant of education having lasting effects on health.^38^^,^^39^ Moreover, the socio-economic mix of schools and the educational expectations within schools may influence social class differences in educational achievement.^40^ Thus, apart from studies focusing on education at individual or interpersonal levels, further research focusing on education at system/organization or community levels is needed. Of note, due to the mandatory minimum age requirements for leaving school and greater availability of higher education, the range in the amount of education obtained within some populations is narrowing.^18^ In HICs at least, with the exception of older cohorts, it is rare to find adults with low or very low education, so shifting the focus towards looking at educational quality and type along with level is needed.

As different education elements may be differentially associated with health outcomes, using conventional measures of education may violate the consistency assumption.^6^ The consistency assumption entails that the exposure (e.g. education) needs to be detailed with enough specificity that any variation within the exposure specification would not result in a different outcome. For instance, literacy may have a different impact on health than the number of years of education and may ultimately lead to different policy recommendations.^6^ Thus, considering several indicators of education allows a better understanding of the context.

### Formal vs informal/experimental education

Formal education measures do not capture informal and non-accredited education, including on-the-job training and other career investments made by individuals with similar levels of formal schooling.^41^ This neglects the concept of life-long learning, which is a dynamic, ever-evolving accumulation of knowledge, skills and capacities. Measures of formal education neglect education at later adulthood and older ages, as formal education is normally completed in young adulthood.

To date, the informal component of education is rarely considered.^27^ This may be due to the complexity of the task of eliciting, coding, classifying and interpreting a wide range of formal and informal educational experiences. Moreover, reverse causality is an issue to be considered when using educational measures that include post-school qualification and training at work rather than measures based on school qualifications.^27^

## Recommendations

**Figure 2:**
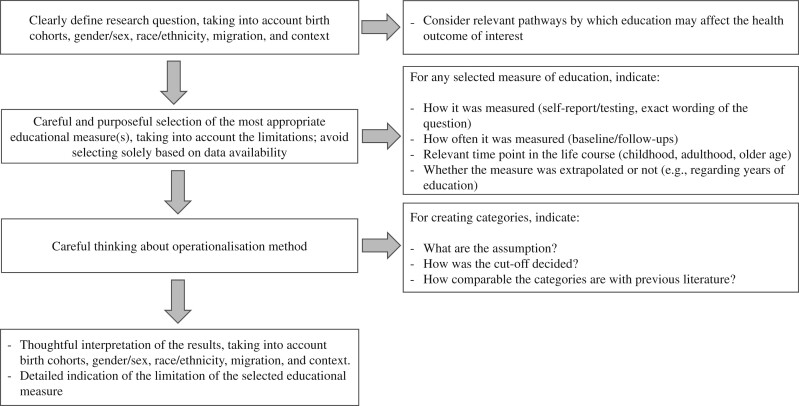
Recommendations for appropriately selecting and operationalizing educational measures in health inequality studies

In Figure 2, we summarize recommendations regarding how to appropriately select the best available educational measure(s) with regard to a research question, considering birth cohorts, social groups and contexts that capture and compare the diversity of participants’ educational experiences over time in health inequality studies. We also recommend considering the quality of education as well as the quantity along with formal and informal education throughout the life course. To do this, cohort studies, public health research and surveillance need to collect data on quality and informal education routinely.

## Conclusions

We have presented conceptual arguments to illustrate major methodological issues and challenges associated with current approaches regarding educational measures in health inequality studies. Consequently, we suggested strategies and recommendations for improving approaches towards selection and operationalization of educational measure(s). We also highlighted the potential future direction of health inequality studies towards including other facets of education and considering the impact of informal education on health. Education plays a potentially important and multifaceted role in redressing health inequalities and by enhancing our research assumptions and methods about hypothesized mechanisms, we may come closer to understanding this in more depth.

## Ethics approval

Not applicable.

## Data availability

Not applicable.

## Author contributions

S.K.S. conceived of the idea for the manuscript. S.K.S. drafted the manuscript. J.M. contributed to manuscript preparation. All authors contributed to critically reviewing and revising the draft. All authors read and approved the final manuscript before submission.

## Funding

S.K.S. is supported by the Australian Research Council Centre of Excellence in Population Ageing Research (project number CE170100005). C.J. received funding from the Institut National du Cancer & the Institut de recherche en santé publique [grant agreement no. (2019–204)]. M.K.I. receives funding from the European Research Council (ERC) under the European Union’s Horizon 2020 research and innovation programme [grant agreement no. (856478)]. The funders had no role in preparation of the manuscript and decision to publish.

## Conflict of interest

None declared.
